# Boundary-Layer Detection at Cryogenic Conditions Using Temperature Sensitive Paint Coupled with a Carbon Nanotube Heating Layer

**DOI:** 10.3390/s16122062

**Published:** 2016-12-03

**Authors:** Kyle Z. Goodman, William E. Lipford, Anthony Neal Watkins

**Affiliations:** 1 Analytical Mechanics Associates, Inc., 18 Langley Blvd., MS 493, Hampton, VA 23669, USA; kyle.z.goodman@nasa.gov; 2 NASA Langley Research Center, 18 Langley Blvd., MS 493, Hampton, VA 23669, USA; william.e.lipford@nasa.gov

**Keywords:** temperature sensitive paint (TSP), carbon nanotubes (CNT), transition detection, cryogenic testing, natural laminar flow

## Abstract

Detection of flow transition on aircraft surfaces and models can be vital to the development of future vehicles and computational methods for evaluating vehicle concepts. In testing at ambient conditions, IR thermography is ideal for this measurement. However, for higher Reynolds number testing, cryogenic facilities are often used, in which IR thermography is difficult to employ. In these facilities, temperature sensitive paint is an alternative with a temperature step introduced to enhance the natural temperature change from transition. Traditional methods for inducing the temperature step by changing the liquid nitrogen injection rate often change the tunnel conditions. Recent work has shown that adding a layer consisting of carbon nanotubes to the surface can be used to impart a temperature step on the model surface with little change in the operating conditions. Unfortunately, this system physically degraded at 130 K and lost heating capability. This paper describes a modification of this technique enabling operation down to at least 77 K, well below the temperature reached in cryogenic facilities. This is possible because the CNT layer is in a polyurethane binder. This was tested on a Natural Laminar Flow model in a cryogenic facility and transition detection was successfully visualized at conditions from 200 K to 110 K. Results were also compared with the traditional temperature step method.

## 1. Introduction

For the validation of aerospace vehicle concepts and prediction tools, it is often desired to test in conditions that most closely resemble those of flight. This is generally performed in a high Reynolds number environment, in which the momentum of the fluid dominates the flow, and turbulent flow is present. High Reynolds number ground-based testing of aerospace models and concepts is often undertaken in facilities capable of operating at cryogenic conditions, in which cooling the test gas to near liquid nitrogen temperatures can achieve Reynolds numbers approaching 500 M/m [1]. One important aspect that is often desired in this type of testing is the knowledge of where the flow transitions on the surface from laminar to turbulent. The determination of this location can be critical for accurate drag estimation, and there are efforts underway to design wing shapes and vehicle concepts that can delay this transition for drag reduction (leading to decreased fuel usage). The location of transition is typically indicated by the change in the adiabatic wall temperature on the surface in areas of laminar versus turbulent flow.

There are several methods to determine transition location in ambient facilities, including multi-element hot-film sensor systems [2,3,4], sublimating chemicals, [5,6], and infrared (IR) thermography [7,8,9]. The multi-element hot-film sensors have been demonstrated down to cryogenic conditions [10]. However, these are point-based measurements that have difficulty providing global measurements on complex models. Both sublimating chemicals and IR thermography can provide these global measurements, but each suffers from distinct disadvantages operating in a cryogenic facility. Sublimating chemicals require frequent access to the model, and to date there are no chemicals that can be applied for cryogenic testing that will not sublimate immediately at ambient conditions. IR thermography can directly image these temperature changes, and measurements at cryogenic conditions have been accomplished using a commercially available IR camera in the 8–12 µm wavelength range [11] and using a specially designed long-wave IR camera (13–15 µm wavelength range) capable of operating at 100 K [12]. However, standard IR thermography suffers from the inherent low amount of IR radiation present at cryogenic conditions, and the custom camera provided relatively small image sizes (128 × 192 pixels) and requires liquid helium cooling of the sensor for operation.

An alternative to these techniques for detecting transition at cryogenic temperatures is based on Temperature Sensitive Paint (TSP) [13,14,15]. TSP is typically composed of a gas impermeable binder in which a luminescent molecule is immobilized [16]. With a suitable binder, changes in the output of the luminescent molecule are due to the changes in the quantum yield due to changes in temperature (i.e., thermal quenching). The relationship between the luminescence of the probe molecule and the absolute temperature generally follows Arrhenius behavior over a certain range [16]

(1) $$\ln\frac{I(T)}{I(T_{REF})}=\frac{E_{NR}}{R}\left(\frac{1}{T}-\frac{1}{T_{REF}}\right)$$

where *E_NR_* is the activation energy for the non-radiative process, *R* is the universal gas constant, and *T_REF_* is the reference temperature. However, for some TSPs, Equation (1) cannot fully describe the behavior, especially outside of temperature ranges where Arrhenius behavior occurs. Thus, it is also common to simply model the behavior of the TSP in a more generalized sense

(2) $$I(T)/I(T_{REF})=f(T/T_{REF})$$

where *f*(*T/T_REF_*) is a function that can be linear, polynomial, exponential, etc., to fit the experimental data over a suitable temperature range. This behavior is dependent on the nature of the probe, thus it is possible to select molecules that can lead to formulations that are temperature sensitive from cryogenic to 473 K [15,16,17,18].

Traditionally, for detecting transition at cryogenic conditions, a temperature step is introduced into the tunnel to enhance the natural temperature change due to transition (depending on flow temperature and local Mach number, this can be on the order of 0.1 K or less). This is usually accomplished by rapidly changing the liquid nitrogen injection rate into the tunnel in either a positive (less nitrogen flow, resulting in a temperature ramp up) or a negative (more nitrogen flow, resulting in a temperature ramp down) direction. While quite effective in increasing the temperature experienced on the model, this can add a significant cost in terms of data acquisition time and facility operation. In addition, there can also be a significant change in the local flow conditions during the step.

Recently, however, work has been presented combining TSP with a carbon nanotube (CNT) based heating layer [19,20]. The CNT heating layer acts as a resistive heater that can locally increase the temperature on the model surface when current is flowed through it. This provides a means to apply a temperature step directly to the model (as opposed to the flow), greatly decreasing the data acquisition time (as the tunnel does not need to recover after each temperature step) and stability in the flow conditions. However, the TSP/CNT system demonstrated degradation and ceased operation at 130 K, [20] most likely due to the fact that the CNT heater layer was based on an acrylic polymer. This paper will present an improvement to the system using a CNT heater layer based on a polyurethane matrix. Laboratory tests have shown that the system is resilient and functional down to 77 K. A natural laminar flow airfoil was also coated with the improved system and transition measurements were conducted in the NASA Langley Research Center (LaRC) 0.3-m Transonic Cryogenic Tunnel (0.3-m TCT) successfully down to 110 K.

## 2. Materials and Methods

### 2.1. Preparation of TSP/CNT System

The TSP formulation used in this work is based on a formulation developed at NASA LaRC. Versions of this TSP have been used for transition detection at cryogenic conditions at the National Transonic Facility (NTF) [21] and for measurement of heating properties at hypersonic conditions [22]. The formulation is based on a polyurethane sealant in which a ruthenium-based luminophore is dissolved. The sealant acts as a gas impermeable binder, and the ruthenium luminophore can be excited using blue lights (e.g., blue LEDs) and exhibits a significant Stokes shift, emitting near 600 nm. This allows easy discrimination of the excitation light from the emitted luminescence using optical filters. For this work, the TSP employed bis-(2,2′,2′′-terpyridine) ruthenium(II) chloride (Ru(trpy)_2_) as the luminophore, which has good sensitivity and luminescence output at cryogenic conditions [13,15,21].

The CNT heater layer consists of carbon nanotubes suspended in a polyurethane base and is commercially available as Carbo E-therm from Future Carbon [23]. This suspension is easy to work with, can be applied using conventional painting techniques, and has shown excellent durability in cryogenic conditions.

The TSP/CNT system is applied to the model surface in several steps. First, an adhesion layer was applied to the surface (~10 µm) and allowed to cure in air. Next, a white polyurethane layer (~50 µm) was applied to act as an insulation layer. This layer can be either air cured overnight or cured at 70 °C for 2 h. After this layer is cured, the electrical connections are applied. For this work, simple copper tape was employed. Then the Carbo E-therm (~50 µm) was applied and allowed to cure in air for about 1 h. Next, another layer of the white polyurethane was applied (~50 µm). This layer is needed as the Carbo E-therm is black in color. The white layer serves to scatter more of the emission light away from the surface for collection by the camera. Finally, the TSP topcoat (~40 µm) consisting of 750 ppm Ru(trpy)_2_ dissolved in a clear polyurethane sealant (0.75 mg Ru(trpy)_2_: 1 g sealant) was applied and allowed to cure. This topcoat can then be sanded for the desired finish. For this test, the roughness (R_a_) was measured to be less than 0.2 µm.

### 2.2. Model and Facility

**Figure 1:**
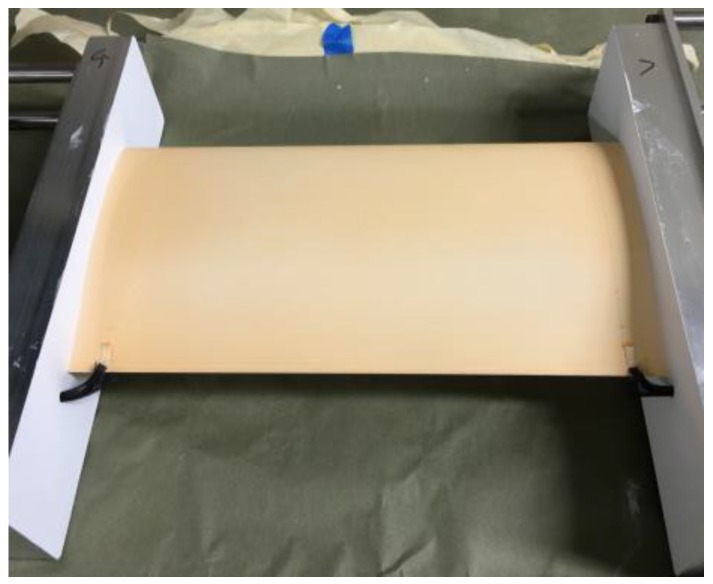
HSNLF(1)-0213 airfoil coated with TSP/CNT.

The wind tunnel testing was performed on a high speed natural laminar flow (HSNLF) wing, HSNLF(1)-0213. A description of the airfoil along with the coordinates are provided by Sewall et al. [24]. The airfoil was constructed from aluminum with a chord of 0.165 m and a span of 0.330 m. The upper surface of the airfoil was coated with the TSP/CNT system and electrical excitation of the CNT layer was provided by parallel conductors placed about 12 mm from the end plates. For this test, the CNT layer was excited from the leading edge to the trailing edge. Connection to the conductors was accomplished using 16 Gauge wire soldered to the end of the conductors. These connections were kept near the end plates at the trailing edge, and their effect on the flow over the surface was minimal. The painted model is shown in Figure 1.

Wind tunnel testing was carried out in the NASA LaRC 0.3-m TCT. The 0.3-m TCT is a continuous-flow, single-return, fan-driven transonic tunnel which can employ either air (ambient temperature testing) or nitrogen (cryogenic temperature testing) as the test medium. It is capable of operating at stagnation temperatures from about 100 K to about 322 K and stagnation pressures from slightly greater than 101 kPa to 607 kPa. Test section Mach number can be varied from near 0 to 0.9. The ability to operate at cryogenic temperatures and high pressure provides an extremely high Reynolds number capability at relatively low model loadings. The test section has computer-controlled angle-of-attack and traversing-wake-survey rake systems. Two inches of honeycomb and five anti-turbulence screens in the settling chamber provide flow quality suitable for natural laminar flow testing. The relevant characteristics for the 0.3-m TCT are shown in Table 1, and additional design features and characteristics regarding the cryogenic concept in general and the 0.3-m TCT in particular can be found previously published works [25,26].

### 2.3. Instrumentation

#### 2.3.1. Illumination

Illumination of the TSP was achieved using commercially available LED arrays (LM2x-400 from Innovative Scientific Solutions, Inc., Dayton, OH, USA). These arrays were designed specifically for PSP and TSP applications, thus are capable of producing a very stable output of more than 3 W with 0.1% drift per hour after warm-up. For this work, the LEDs were configured to emit at 460 nm (30 nm bandwidth at full width at half maximum (FWHM)).

#### 2.3.2. Image Acquisition

Images of the TSP output were acquired from a single camera that was placed coincident with the LED arrays. The camera employed was a PSP-CCD-M (Innovative Scientific Solutions, Inc., Dayton, OH, USA), having a resolution of 1600 × 1200 pixel resolution operating at either 12-bit or 14-bit digital resolution. The camera was interfaced to the computer via gigabit Ethernet (GIG-E) and capable of acquiring data up to 44 frames per second. For some laboratory testing, an infrared camera (SC-6701, FLIR, Nashua, NH, USA) was employed to monitor heating from the CNT heater.

#### 2.3.3. Illumination and Image Acquisition Mounting in 0.3-m TCT

**Figure 2:**
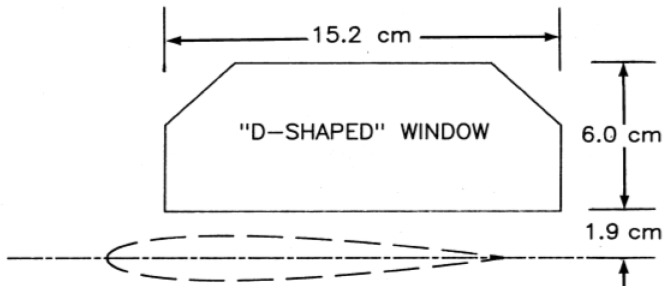
Geometry of the “D-shaped” window with a generic airfoil showing approximate location. From [27].

**Figure 3:**
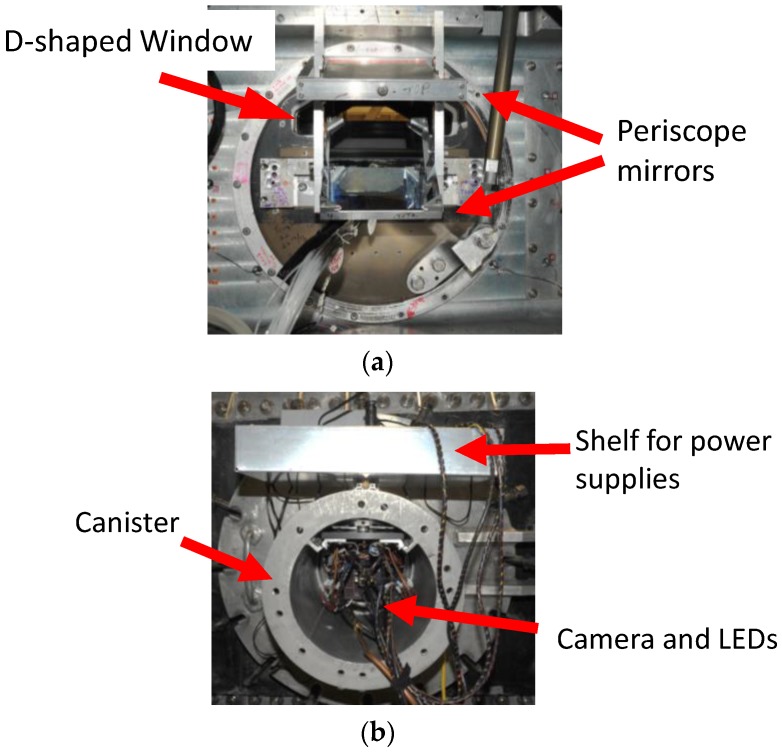
Mounting of equipment at 0.3-m TCT: (**a**) optical setup showing the “D-shaped” window and the periscope assembly; and (**b**) canister mounted onto the side of the tunnel containing the camera and LED illumination sources.

The optical access for the 0.3-m TCT consists of a “D-shaped” window that was originally designed for off-body flow visualization studies. The D-shaped window is constructed of Schlieren quality fused silica that is mounted in the upper half of the circular angle-of-attack turntables. For this work, the airfoil is centered horizontally in the test section with its center-line 1.9 cm below the lower edge of the window. As such, there is no direct optical access to the surface. A diagram of the D-shaped window with a generic airfoil is shown in Figure 2 [27]. In addition, the D-shaped window (and test section) is separated from the outside of the tunnel by a rectangular pressure plenum. To facilitate illumination and image acquisition, a pair of mirrors was deployed as a periscope to allow optical access to the upper surface of the model. This periscope was attached to the test section door and inside the plenum. A photograph of the optical setup is shown in Figure 3a.

Optical access from outside of the plenum is provided by a window placed in the plenum wall. This window is also of Schlieren quality fused silica with a diameter of 22.9 cm. To keep the outer window clear of condensation (due to the large temperature difference on either side of the window), a large canister with a purge ring is connected to the plenum. The camera and the LEDs were placed in this canister. The canister mounted to the plenum is shown in Figure 3b.

#### 2.3.4. Data Acquisition in the 0.3-m TCT

As mentioned above, for transition detection at cryogenic conditions using TSP, it is generally desirable to enhance the natural transition temperature by the introduction of a temperature step. For the TSP/CNT system, this was accomplished by applying a current to the CNT layer and data acquisition generally proceeded using the following paradigm:

1. The flow conditions of the tunnel were established.
2. After stabilization, a series of images was acquired to act as reference images.
3. Current was applied to the heater layer using a remotely operated programmable DC power supply.
4. Images were collected for several seconds during heating (*Temperature Images*).
5. The current was removed and the model was prepared for the next point.

For the data points in which a temperature step was applied by modifying the liquid nitrogen injection, the paradigm was modified as follows:

1. The flow conditions of the tunnel were established.
2. After stabilization, a series of images was acquired to act as reference images.
3. The nitrogen flow was rapidly increased to lower temperature. Meanwhile, image collection from the camera was begun.
4. Images were collected for several seconds throughout the temperature step (*Temperature Images*).
5. The tunnel was reconditioned to match the desired flow conditions. After re-stabilization, the model was prepared for the next point.

## 3. Results and Discussion

### 3.1. Laboratory Testing

Several experiments were performed in the laboratory to verify the ability of the CNT heater to function properly at cryogenic conditions as well as ensure that the TSP could function properly over the CNT heater. Several of these experiments were performed using an IR camera to visualize temperature distribution as well as verify the TSP operation.

**Figure 4:**
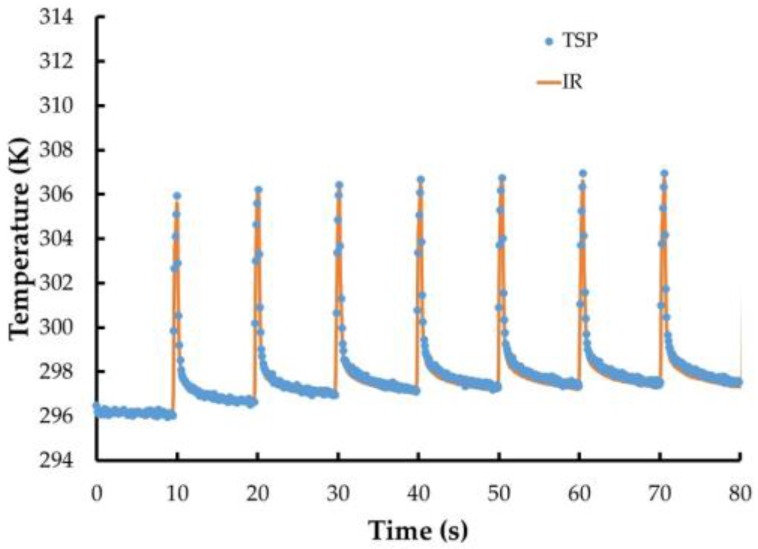
Temperature from TSP (points) and IR camera (line) with 115 W (50 V, 2.3 A) applied to CNT layer.

The first experiment involved painting a small coupon (diameter ~7.6 cm) with the entire system. For this experiment, instead of Ru(trpy)_2_, the TSP used tris(bipyridine)ruthenium (II) chloride (Ru(bpy)_3_). This luminophore displays good luminescence at ambient temperature and has been used in TSP formulations up to 353 K. Illumination of the coupon was accomplished using 460 nm LEDs and TSP images were acquired using the PSP-CCD-M camera. In addition, IR images were also acquired. The resistance measured of the coating was measured as 22 Ω, and several levels of current were applied in a pulsed manner. The temperature calculated from the TSP measurements compared with the IR camera results are shown in Figure 4, with 50 V applied (2.3 A, 115 W). As can be seen, the temperature measured using the TSP coating tracks closely with the temperature measured using the IR camera.

**Figure 5:**
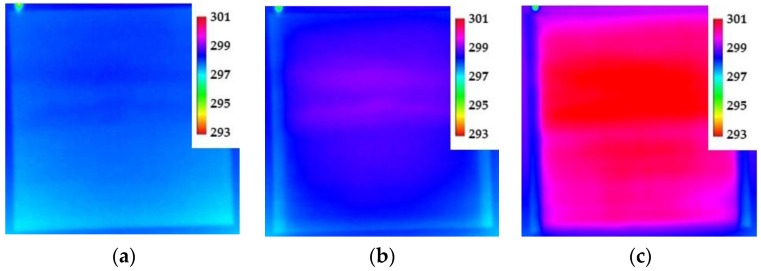
IR images from a larger panel painted with a CNT heater layer: (**a**) No current applied; (**b**) 70 W (32 V, 2.2 A); and (**c**) 385 W (74 V, 5.2 A). Color scale is temperature (K).

The next experiment was to determine if a large sized area could be adequately heated using the CNT heater. A larger sized aluminum plate was coated with the CNT heater layer (no TSP) so that an area approximately 0.93 m^2^ was to be heated. The resistance of the CNT coating for this plate was approximately 14 Ω, and several levels of current were applied. A set of IR images with no current applied, 32 V (2.2 A, 70 W) applied, and 74 V (5.2 A, 385 W) applied are shown in Figure 5. As can be seen, the temperature field is fairly evenly distributed, though there are some bands present near the middle of the plate. This is from an uneven application of the CNT heater layer and shows that care must be used to ensure an even coating. Furthermore, the uneven application of the CNT layer has caused a slight change in either the emissivity of the coating or in its reflectivity, leading to the appearance of the bands even with no current applied. This application of the CNT layer was corrected in the wind tunnel tests.

**Figure 6:**
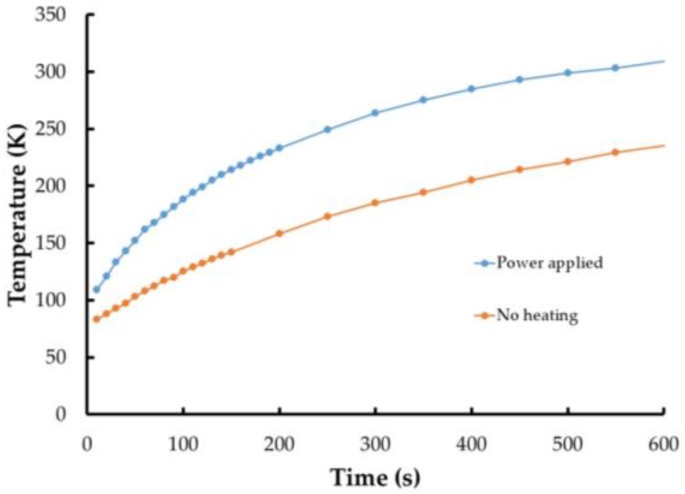
Thermocouple measurements from coupon with CNT heater powered (blue) and unpowered (orange) after immersion in liquid nitrogen.

After verification that the TSP can work with the CNT heating layer and that a larger size (similar to the airfoil size) can be effectively heated, experiments were performed to determine how well the system would operate at cryogenic temperatures. Initially, a small coupon was coated with the system and instrumented with a K-type thermocouple. The entire coupon was immersed into a dewar of liquid nitrogen until the thermocouple reading measured 77 K (the boiling point of liquid nitrogen). Then 100 V (2.2 A, 220 W) was applied to the coupon was applied in 2 s bursts at intervals of 5 s (2 s on followed by 5 s off) with the thermocouple readings constantly recorded. Figure 6 shows the results of this compared with the same coupon cooled to 77 K with no current applied to the heater layer. It is readily apparent that even with these short current bursts, the heater is functioning down to at least 77 K. Additionally, no physical degradation of the paint system was evident even after several repeated cycles of liquid nitrogen immersion followed by rapid warm up to room temperature.

**Figure 7:**
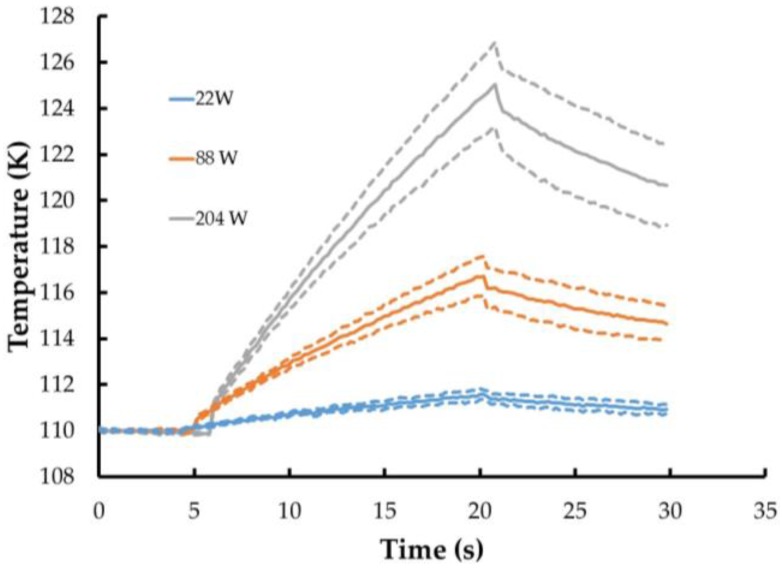
Temperature measured using TSP and CNT heater layer at 110 K.

The final laboratory verification of the TSP/CNT system involved coating the large piece of aluminum described above with the TSP and CNT coatings and imaging the TSP at cryogenic conditions. This is to verify that the heat provided by the CNT layer is sufficient to overcome the convection that will be present at full cryogenic conditions (110 K). This testing was performed in a larger cryogenic chamber that is capable of being cooled to at least 110 K. Unfortunately, a flow field could not be established in the chamber, so these were simply static tests. The change in temperature over the surface during temperature steps initiated with different power to the CNT heater layer is shown in Figure 7. The temperature was measured at five distinct locations on the surface and the average is presented. These show that even with the application of relatively low power (22 W), a measurable change in temperature is achievable. As with the previous testing, the standard deviation across the surface (indicated by the dashed lines) is most likely due to uneven application of the CNT layer and was not present during wind tunnel testing.

### 3.2. Wind Tunnel Testing at 0.3-m TCT

For all wind tunnel testing, registration marks were applied to the model surface and used to map the image data to a mesh of the surface using a direct linear transformation method. More information on this method can be found in Liu and Sullivan [28]. The testing took place over a period of 12 days and the model was not repainted. The resistance of the CNT layer was measured to be ~30 Ω and showed very little deviation throughout the test. All data presented from the test have been calibrated to temperature, with darker regions indicating lower temperature and lighter regions indicating higher temperature. In addition, the flow is from left to right in all images.

#### 3.2.1. Verification of TSP/CNT System in the 0.3-m TCT

**Figure 8:**
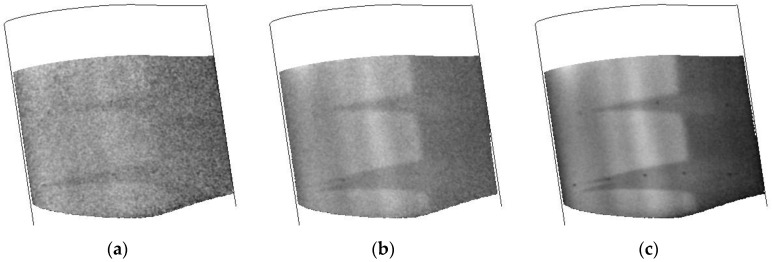
Applying increasing current to the CNT layer: (**a**) 50 V; (**b**) 100 V; and (**c**) 150 V.

Initial testing of the TSP/CNT system in the 0.3-m TCT was accomplished under relatively benign conditions of Mach 0.7 and a temperature of 200 K. For these runs, the Reynolds number was 32.8 M/m. The evaluation consisted of several different runs at a fixed angle of attack (−2°) and applying different current to the CNTs. A comparison of these results is shown in Figure 8. These data were taken approximately 8 s after the application of the current to the CNT layer. The transition point between laminar and turbulent flow is indicated by a sudden change in temperature. As the CNT layer heats the model surface, areas of the surface under laminar flow will be warmer than areas under turbulent flow since the turbulent flow interacts with the surface more, thus cooling it to a greater degree. In these cases, lighter areas represent laminar flow and darker areas represent turbulent flow. As can be seen in Figure 8, even the application of relatively low power (50 V, 83 W) results in a slight increase in the model temperature (~0.5 K) and ~0.1–0.2 K temperature change from laminar to turbulent flow. As the power applied to the CNT layer is increased, this contrast becomes much greater. The transition front is clearly visible, and the wedges that are formed are due to small imperfections on the TSP surface. The greatest temperature increase on the model was ~5 K and a change in temperature from the laminar to the turbulent flow of ~2 K.

**Figure 9:**
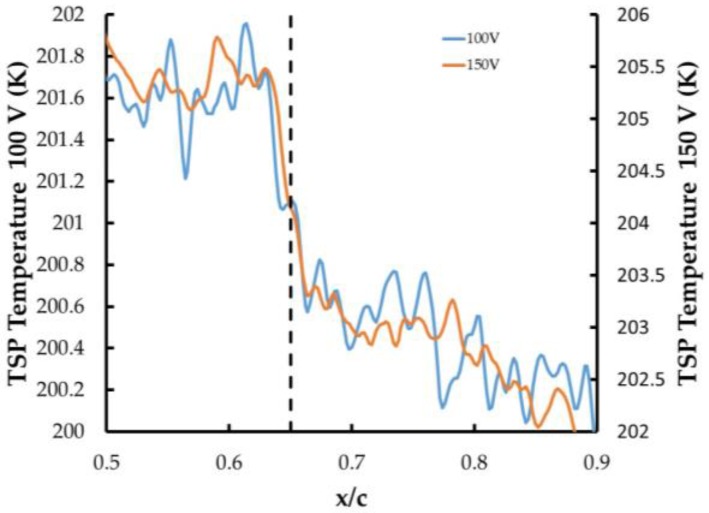
Transition location determined by exciting the CNT layer with 100 V (left axis) and 150 V (right axis).

Recent studies by Constantini et al. [29,30] have shown that care must be taken when interpreting transition results obtained using TSP and applying a temperature step. Their work has shown that when the surface temperature (T_w_) is greater than the adiabatic–wall temperature (T_aw_), then the transition location can vary depending on the T_w_/T_aw_ ratio. To see if the application of different voltages to the CNT layer can cause a change in the transition position, an analysis of the transition location was carried out for each case using a similar procedure outlined by Constantini et al. [29] and the results are shown in Figure 9. For this work, the transition position when 50 V was applied was difficult to visualize accurately. For the 100 V and 150 V case, the data were plotted on different temperature scales to normalize them. As can be seen, the transition location for both cases was at an approximate location of 65% chord. A similar analysis was done with time and no change in the location was observed. This model did not have pressure instrumentation, and there was no way to visualize the lower surface, so it cannot be determined if transition on the lower surface occurred or how stable it was. For this model, however, it was established that the CNT operating voltage did not have a significant effect on the transition location on the upper surface.

**Figure 10:**
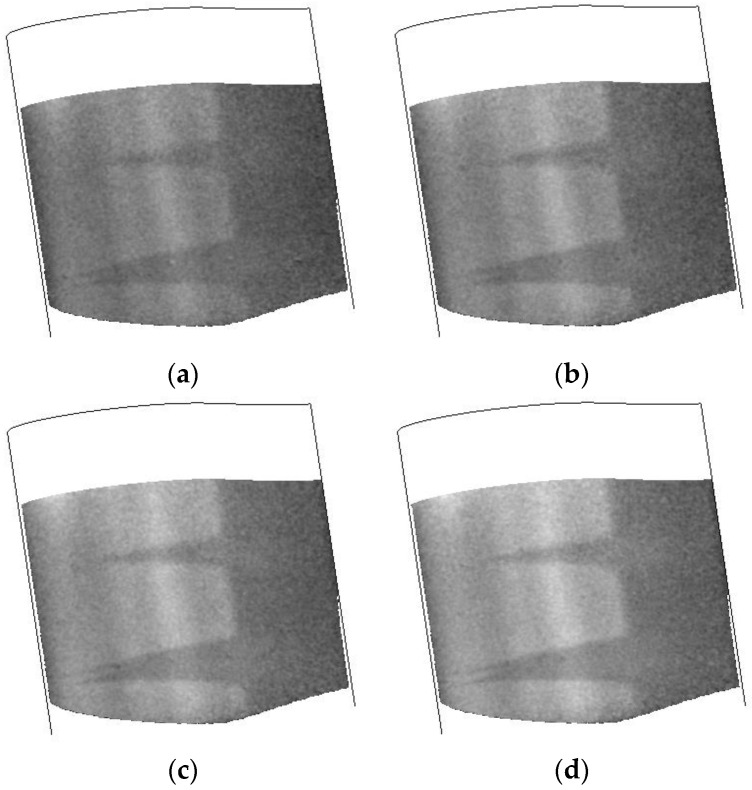
Temperature images collected during a CNT heating cycle (100 V). Data were collected at T = 200 K, Mach 0.7, Re = 32.8 M/m: (**a**) 1.5 s; (**b**) 3 s; (**c**) 4.5 s; and (**d**) 6 s. All times are after start of the heating cycle.

The stability of the TSP/CNT layer is shown in Figure 10. In this experiment, a series of images was acquired after turning on the CNT layer and collected for approximately 10 s. The response of the TSP is presented at several times in Figure 10 and shows that the transition front position and the turbulent wedges are extremely stable, with greater contrast between laminar and turbulent regions realized as the CNT layer is powered for longer times.

#### 3.2.2. TSP/CNT System Response at Different Conditions

Throughout this wind tunnel entry, several different tunnel conditions were employed with this airfoil allowing for a comparison of different tunnel conditions. While there was an aerodynamic component to this test, this paper is concerned chiefly with the operation of the TSP/CNT system. Thus, all data presented here are at an angle of attack at −2°.

**Figure 11:**
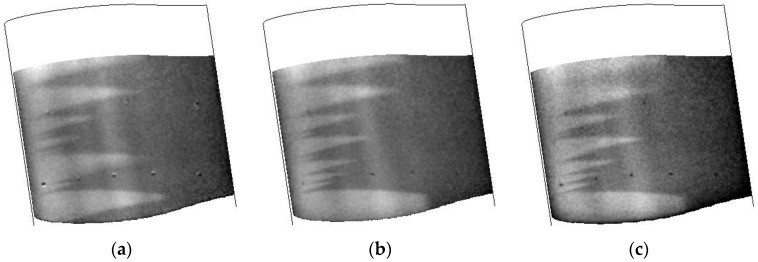
Temperature images collected at different tunnel temperatures during a CNT heating cycle. Data were collected at Mach 0.3, Re = 32.8 M/m: (**a**) 110 K; (**b**) 172 K; and (**c**) 200 K.

The response of the airfoil to different temperatures while maintaining a constant velocity and Reynolds number are shown in Figure 11. While the surface finish did have some issues causing a number of turbulent wedges, the positions of the wedges seem quite consistent. It should be noted that these data were collected on the second day of testing after the tunnel had been warmed. Inspection of the model after the second day showed the presence of small defects and possible oil stains on the leading edge. This is most likely due to debris in the tunnel that is stirred up during tunnel conditioning and running as the number of wedges increased during the typical run schedule. Towards the bottom of the images, it does seem to show the transition front. This shows that the TSP/CNT layer is consistent over the temperature ranges that were studied, including the lowest temperature capable for the facility (110 K).

**Figure 12:**
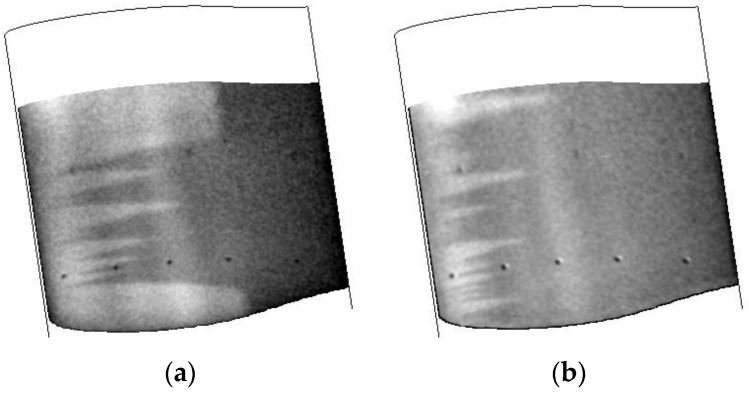
Temperature images collected at different Reynolds numbers. Data were collected at T = 200 K, Mach 0.7: (**a**) Re = 32.8 M/m; and (**b**) Re = 49.2 M/m.

A comparison of the transition behavior at different Reynolds numbers is shown in Figure 12. For this case, the speed of the tunnel was constant at Mach 0.7 and the Reynolds number was changed from 32.8 M/m to 49.2 M/m. At the lower Reynolds number condition, even with the wedges, the transition front is well established. However, as the Reynolds number is increased, the number of wedges also greatly increases, again due to imperfections in the surface finish and the decrease in boundary layer thickness with increasing Reynolds number.

#### 3.2.3. Effect of Surface Finish

While it has been demonstrated that the TSP/CNT system is capable of providing a temperature step on the model surface sufficient enough to determine flow transition location using TSP, the surface finish of the TSP layer was a limiting factor for full surface characterization. The initial finish of the TSP coat was ~0.2 µm after sanding. This finish was sufficient to observe the transition from laminar to turbulent flow in many of the cases. However, turbulent wedges were evident in most of the images. Many of these wedges did not seem to originate from the leading edge, thus it is reasonable to conclude that these are most likely from small imperfections on the TSP surface. Additionally, as shown in the time series of experiments, the positions of the wedges were highly consistent throughout the run.

After the first days of running, the model was removed to install a flow control concept for testing. This concept is beyond the scope of this paper, but for the transition work, it served as a (rather large) distributed roughness element that would transition the flow in a known location. Additionally, the TSP surface was further sanded and a thin layer of wax was added to fill in the small imperfections on the surface. After this treatment, the surface roughness was reduced to ~0.15 µm. Several additional runs were made at 200 K.

**Figure 13:**
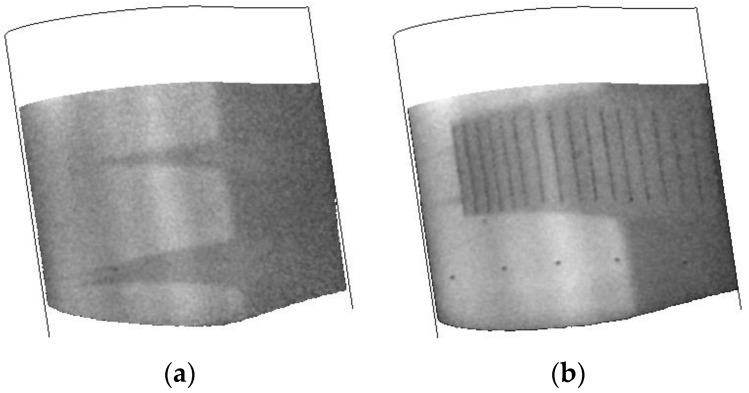
Results of surface finish comparing before and after a sanding and waxing step. Data were collected at T = 200 K, Mach 0.7, Re = 32.8 M/m: (**a**) before sanding and waxing; and (**b**) after sanding and waxing.

The effect of the reduction in the surface roughness is shown in Figure 13. This was a repeat of the testing shown in Figure 10 above. As can be seen, the transition front is easily visualized and the turbulent wedges that were present in Figure 10 have now been removed. The flow control concept is acting as a large roughness element that has tripped the laminar flow to turbulent. Not only has this shown the need to ensure the surface finish is as smooth as possible, it also demonstrates the robustness of the TSP/CNT system in that it could be warmed to room temperature, the surface significantly altered (with the addition of the flow control concept and further polishing), and the performance does not degrade. This can have big implications for transition to a larger facility with respect to installation as well as durability.

#### 3.2.4. Comparison with the Traditional Temperature Step Method

To complete the feasibility test of the TSP/CNT system, its performance was compared with the traditional method of introducing the temperature step in the 0.3-m TCT. This method involves the injection of liquid nitrogen at a rate sufficient to introduce a rapid change in temperature over the model [14]. For this testing, the tunnel was maintained at a constant temperature before the step (200 K), constant velocity (Mach 0.7), constant angle of attack (−2°), and an initial Reynolds number of 32.8 M/m. For these runs, tunnel conditions were continuously recorded at a rate of 20 Hz.

**Figure 14:**
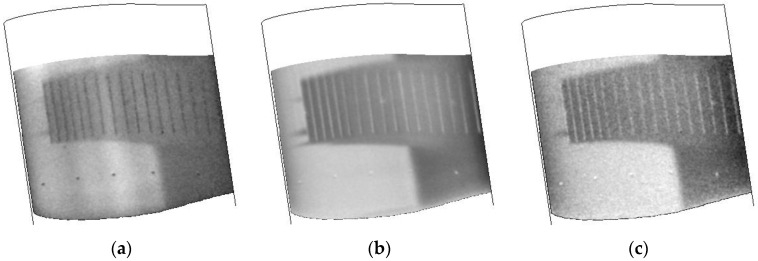
Effect of the method for introduction of the temperature step. Data were collected at T = 200 K, Mach 0.7, Re = 32.8 M/m: (**a**) CNT heater; (**b**) fast injection; and (**c**) slow injection.

A comparison of the results obtained with the TSP/CNT system and obtained with two different injection rates (the fastest and the slowest) are shown in Figure 13. For Figure 13, the TSP/CNT image shown was collected ~8 s after application of current to the CNT layer. For the fastest injection rate, the image shown was acquired ~10 s after the start of the injection. For the slowest injection rate, the image shown was acquired ~35 s after the start of the injection. As can be seen, in all cases, the transition front is easily seen. The temperature change on the surface and at transition for each image in Figure 14 is listed in Table 2, showing similar temperature changes measured using each technique. As expected, the rapid liquid nitrogen injection method provides a better contrast image with lower noise than the other techniques, but all would be viable for the intended purpose of determining the location of transition.

**Figure 15:**
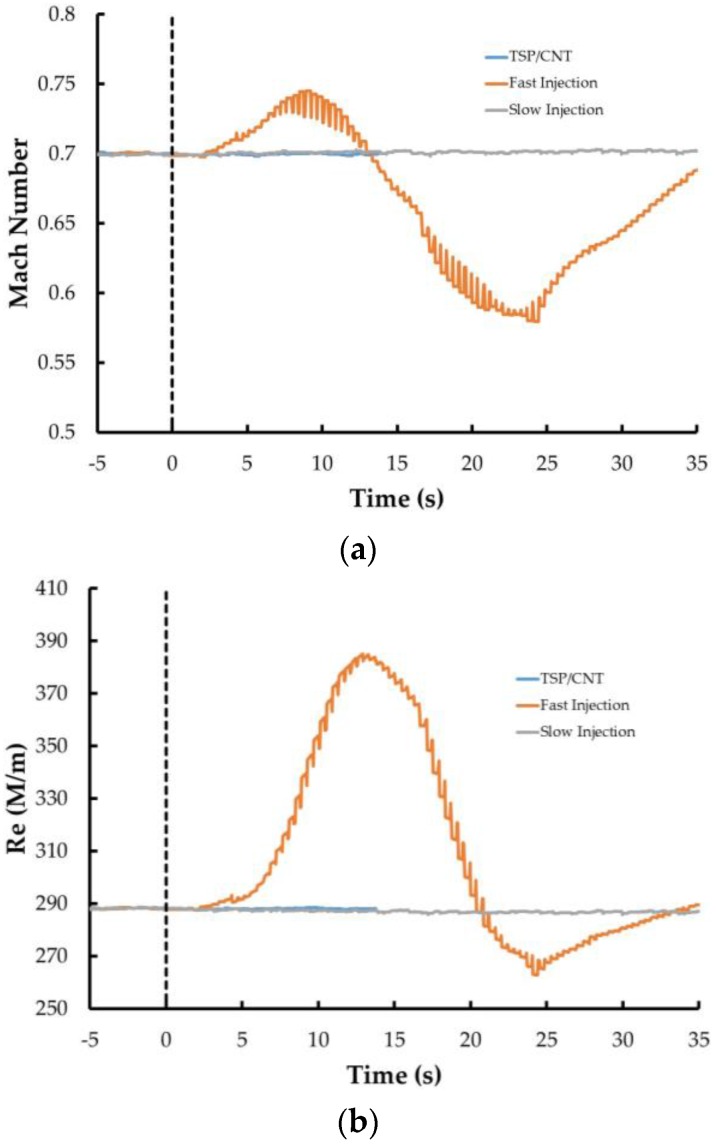

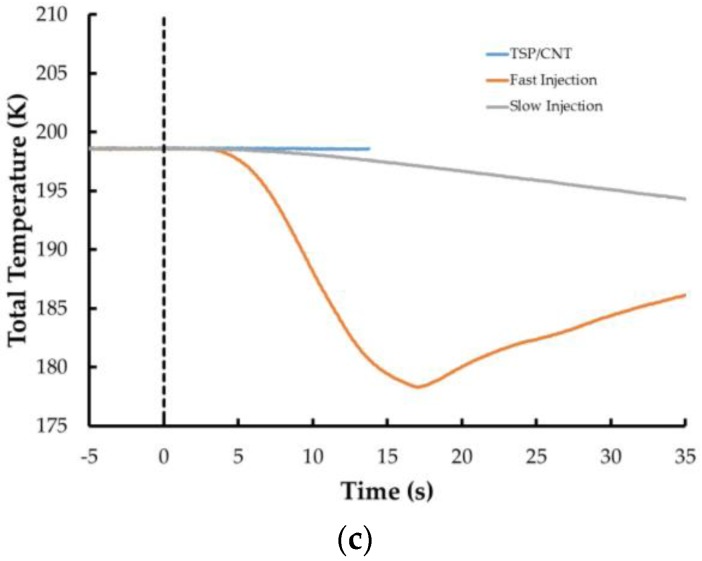
Tunnel conditions during the temperature step introduced using the different methods. The start of the injection is marked at t = 0 s: (**a**) Mach number; (**b**) Reynolds number; and (**c**) total temperature.

**Figure 16:**
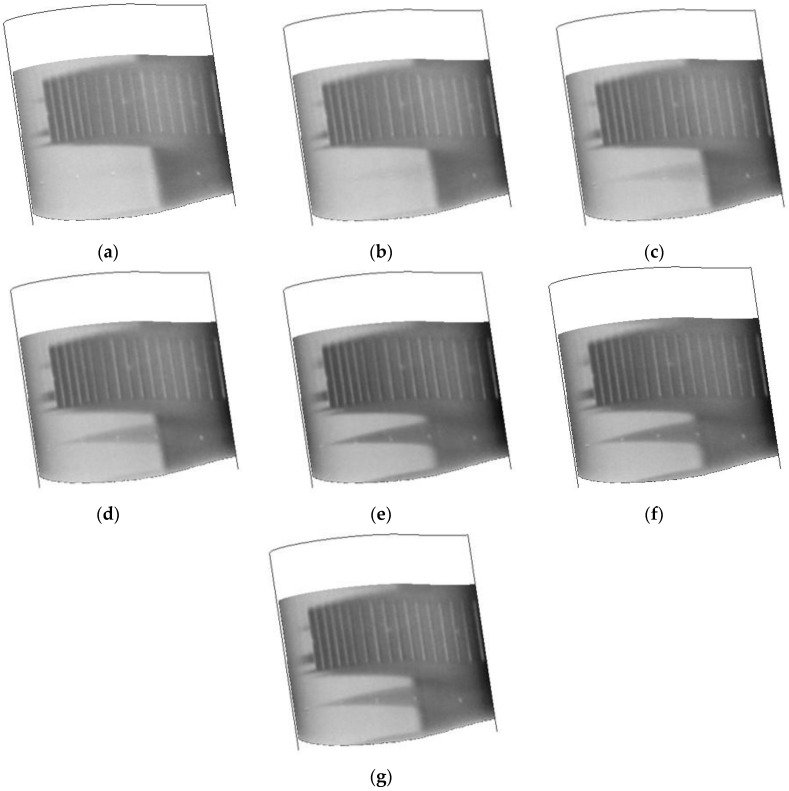
Temperature images collected after introduction of a temperature step using the fast injection method. Tunnel conditions are the same as Figure 13: (**a**) 10 s; (**b**) 10.4 s; (**c**) 10.8 s; (**d**) 11.2 s; (**e**) 11.6 s; (**f**) 12 s; and (**g**) 12.4 s.

One of the advantages of the TSP/CNT system is that the temperature step is applied directly to the model as opposed to the tunnel flow. This should result in greater stability of the tunnel conditions during the temperature step. Several different tunnel parameters were collected during the run with the TSP/CNT layer as well as the nitrogen injection methods. A comparison of the Mach number, Reynolds number, and total temperature are shown in Figure 15. For each case, the time scale is adjusted so that the introduction of the temperature step (either by CNT or by liquid nitrogen) is set to t = 0. When the fast injection method was employed, the tunnel conditions changed dramatically, especially in regards to Mach number and Reynolds number. After about 10–12 s, the flow in the tunnel began to choke, causing the tunnel to move into a recovery mode. The effects of the rapidly changing tunnel conditions on the transition are shown in the time series of images shown in Figure 16. These are sequential images starting at 10 s after the temperature step is initiated. As can be seen, the initial image shows a good transition front with only small turbulent wedges in front of the roughness element. However, in the next image, a bigger wedge is starting to form, and is fully established soon after. In addition, there appears to be a very slight systematic shift in the position of the transition front of ~2%–3% during the series. This emphasizes that if a rapid temperature step is to be employed, care must be taken when these data are evaluated and that the tunnel conditions will most likely change significantly.

For both the slow injection method and the CNT heating method, no change in the Mach number or Reynolds number is observed (the lines are practically overlapping). However, the total temperature of the tunnel decreases with the slow injection method as opposed to the CNT heating method, and it requires a longer amount of time to establish a suitable temperature step on the model to acquire better quality images. Overall, the TSP/CNT method showed a conservative increase in efficiency of at least a factor of 5 compared with the temperature steps. This is taking into account the time necessary to establish the temperature gradient as well as the time needed for the tunnel to re-equilibrate after performing the liquid nitrogen injection and prepare for the next data point. This can have very significant implications in larger facilities. It is conceivable that with the TSP/CNT method, data points (including both reference images and temperature images) could be acquired in only a few seconds in these facilities. Previous testing in larger facilities has shown that it could take more than a minute to acquire a single data point using the liquid nitrogen injection methods [15,31].

## 4. Conclusions

This paper has demonstrated a system using TSP coupled with a CNT heater for the detection laminar to turbulent flow in a cryogenic facility. This system works by inducing a temperature step on the model surface to enhance the natural temperature change when flow transitions from laminar to turbulent. While the concept has been successfully demonstrated previously in several facilities, its success has been limited at the lowest temperatures employed for full flight Reynolds number testing (110 K and below) due to degradation of the acrylic binder used in the CNT layer. The TSP/CNT system demonstrated here employed a CNT layer based on a polyurethane binder as opposed to an acrylic binder. This system was validated in the laboratory and successfully operated down to temperatures of 77 K. Methodologies for applying it to larger scale surfaces were developed and the concept was successfully demonstrated in the 0.3-m TCT facility down to 110 K with no physical degradation of the system observed. The system was also robust enough to be handled after application, including further smoothing of the surface to improve the quality of data. The performance of the TSP/CNT system was also compared with the traditional method of introducing a temperature step by changing the injection rate of liquid nitrogen. While the liquid nitrogen injection methods can provide larger temperature steps (and thus higher contrast images), care must be taken to ensure that the liquid nitrogen injection rate does not significantly alter the tunnel conditions. This can cause changes in the transition front location as well as induce turbulent wedges on the surface. Even if the injection rate is slow enough to maintain constant tunnel conditions, the TSP/CNT layer can typically provide quality data in a shorter time frame as the temperature step on the model can be applied more quickly than the slow temperature step in the tunnel. More research into application methodologies and other limitations are currently being pursued, but the TSP/CNT system has the promise of greatly increasing the efficiency of transition testing at cryogenic facilities, resulting in significant savings (in both cost, energy, and time).

## Figures and Tables

**Table 1:** Relevant characteristics of the 0.3-m TCT.

Test Section Dimensions	0.33 m by 0.33 m
Speed	Mach 0.1 to 0.9
Reynolds Number	3.3 to 330 M/m
Stagnation Temperature	100 to 322 K
Stagnation Pressure	101 to 607 kPa
Test gas	Nitrogen or air

**Table 2:** Temperature changes on the model surface for conditions shown in Figure 13.

Temperature Step Method	Maximum ΔT on Model	ΔT Transition (Laminar to Turbulent)
TSP/CNT	4 K	2 K
Rapid liquid nitrogen injection	−8 K	−4 K
Slow liquid nitrogen injection	−2.5 K	−2 K
